# The Role of the Immune Response in Brain Metastases: Novel Imaging Biomarkers for Immunotherapy

**DOI:** 10.3389/fonc.2021.711405

**Published:** 2021-10-26

**Authors:** Rasheed Zakaria, Mark Radon, Samantha Mills, Drew Mitchell, Carlo Palmieri, Caroline Chung, Michael D. Jenkinson

**Affiliations:** ^1^ Department of Neurosurgery, University of Texas M.D.Anderson Cancer Center, Houston, TX, United States; ^2^ Faculty of Health and Life Sciences, University of Liverpool, Liverpool, United Kingdom; ^3^ Department of Radiology, Walton Centre NHS Foundation Trust, Liverpool, United Kingdom; ^4^ Department of Imaging Physics, University of Texas M.D.Anderson Cancer Center, Houston, TX, United States; ^5^ Department of Radiation Oncology, University of Texas M.D.Anderson Cancer Center, Houston, TX, United States; ^6^ Department of Neurosurgery, Walton Centre NHS Foundation Trust, Liverpool, United Kingdom

**Keywords:** immune response, brain metastasis (BM), microenvironment, immunotherapy, biomarkers, MRI, PET

## Abstract

Brain metastases are a major clinical problem, and immunotherapy offers a novel treatment paradigm with the potential to synergize with existing focal therapies like surgery and radiosurgery or even replace them in future. The brain is a unique microenvironment structurally and immunologically. The immune response is likely to be crucial to the adaptation of systemic immune modulating agents against this disease. Imaging is frequently employed in the clinical diagnosis and management of brain metastasis, so it is logical that brain imaging techniques are investigated as a source of biomarkers of the immune response in these tumors. Current imaging techniques in clinical use include structural MRI (post-contrast T1W sequences, T2, and FLAIR), physiological sequences (perfusion- and diffusion-weighted imaging), and molecular imaging (MR spectroscopy and PET). These are reviewed for their application to predicting and measuring the response to immunotherapy in brain metastases.

## Introduction

The overall clinical burden from brain metastases (BM) is increasing, most likely to due to more widespread use of brain imaging, even in asymptomatic patients, and improved control of extracranial disease and survival in cancers that predispose to BM. Incidence increases with age and varies with the primary, being most common in non-small-cell lung cancer, breast cancer, and malignant melanoma. This has led to BM occupying substantially more of the neurosurgery, radiology, and oncology workload compared to other brain tumors in recent years (1).

Immunotherapy is a transformative field of treatment for cancer and encompasses a variety of therapeutics including vaccines, oncolytic viruses, cell-based therapies, and immune checkpoint inhibitors (ICI). A number of trials of ICI for solid organ cancers that have included patients with BM suggest a heterogeneous but robust response in the brain [(2) summary (3), for specific example in metastatic melanoma]. This has come on the background of increased investigation of the BM microenvironment and the understanding that this is an immunologically distinct rather than immune-isolated compartment (4, 5). The immune response to BM therefore requires further investigation, to elucidate both the underlying mechanism of this response and that of resistance to therapeutics. Imaging is frequently used in the diagnosis and management of BM, so it is logical that brain imaging techniques are used to investigate the immune response and as a possible source of biomarkers of the immune microenvironment in these tumors (6–9).

At the time of writing this report, there was insufficient data in this field for a systematic review applying PRISMA (10) guidelines; therefore, we have performed a narrative review and categorized the *clinically available* techniques in brain imaging—taking in studies from other brain tumors and therapies—to assess the prospects for the development of biomarkers of response to immunotherapy in BM.

## Conventional MRI Biomarkers of Early Treatment Response

The radiological evaluation of BM response to therapy has fundamentally relied on tumor size on T1-weighted (T1W) contrast-enhanced MRI. Within the context of clinical trials—the Response Assessment in Neuro-Oncology Brain Metastases (RANO-BM) group guidance of 2015—up to five target lesions are identified and measured; these ideally should be large, easily evaluated, and not pretreated (11). There are a variety of definitions of what constitutes a measurable target lesion, but the group suggested these should be at least 10 mm in diameter. The intracranial response was recognized as being independent of extracranial response and accounts for this measured size plus clinical condition and corticosteroid dose. From a radiological perspective, the biomarker here is simply the tumor diameter in its longest axis. A percentage decrease (30% or more for partial response, 100% for complete response) or increase (20% or more for progressive disease) of the summed diameters is used as a surrogate of the true, unmeasurable biological disease response to therapy.

These current RANO methods are two-dimensional, but volume of disease may replace size for a variety of reasons in the future (12). Regarding thresholds, a volumetric change of 20% appears to be a reproducible figure between different readers and may be associated with neurological improvement (13, 14), although the RANO-BM group was more conservative and suggested a 65% volume decrease (corresponding to a 30% diameter reduction, subject to the assumption of a spherical lesion) as a safer cutoff for defining a partial response to therapy (11). In summary, tumor volume is not part of standard clinical reporting to assess response at present, but is useful because of emerging evidence it may better reflect prognosis compared to two-dimensional measurements and is an important metric to include in clinical trials.

**Figure 1:**
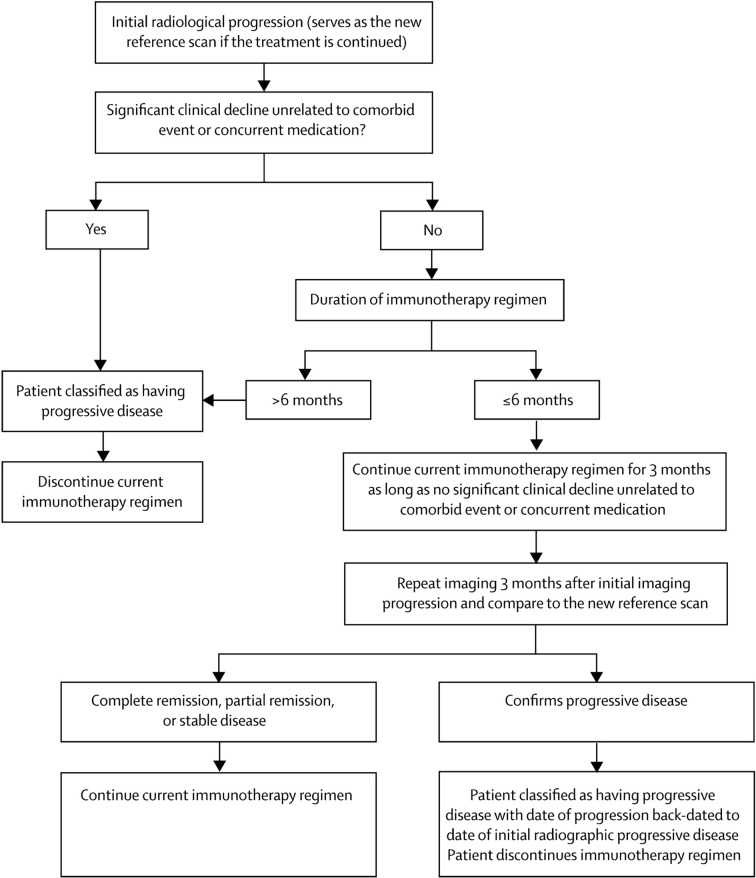
Suggested algorithm for evaluation of progressive imaging findings among neuro-oncology patients undergoing immune-based therapies. Reproduced under Creative Commons license without modification (http://creativecommons.org/licenses/by/4.0/) from (19).

The challenge with measuring sizes—either diameter or volume—is that immunotherapy-induced inflammation may mimic progression radiologically. This was already well documented in glioma as the “flare phenomenon,” and in early immunotherapy trials, some extracranial metastases increased in size due to immune infiltration, or new areas of enhancement appeared whilst a response was mounted but disappeared later as there was no viable tumor there (15, 16). Despite these potential pitfalls, conventional RANO-BM was applied to an early ICI trial of pembrolizumab for non-small-cell lung cancer (NSCLC) and melanoma BM (there was no stratification by genetic mutations such as EGFR and BRAF), and the authors found good concordance with other response criteria, although noted that lowering the cutoff for measurable lesions to 5 mm would have included more patients (17). To address this issue of inflammation and size, the immune related Response Criteria (ir-RC) were devised by a different panel of experts for solid tumors (18). As immunotherapy became more widely applied to patients with BM (and glioma), these were reconsidered for neuro-oncology alongside the original RANO and RANO-BM criteria to generate the immunotherapy or iRANO criteria, which are summarized in **Figure 1** (19). Based on the available evidence, this group determined that major radiographic changes occurring after 6 months following the start of immunotherapy are likely to be progression, but *until this time* there should be two major differences in approach compared to other therapies in neuro-oncology. First, in patients with no significant clinical decline, new enhancing lesions should not define progressive disease, on the basis that they may represent inflammation that subsequently resolves. Second, in patients with no significant clinical decline, rather than obtaining a confirmation scan 4 weeks after the initial imaging that suggests progression, this should be done after 3 months to allow time for inflammatory changes to occur and potentially resolve. As in the original criteria, confirmation of progression is backdated to the initial scan that suggested this. The caveat was that the patient in both circumstances must be clinically well, with no new or worsened deficits (unless such deficits have a specific cause like medication or a comorbid event). Finally, the role of steroids in patients with BM undergoing immunotherapy is more complex than in other therapies as they may dampen the immune response and yet may be required to manage symptoms. Therefore, the group deemed that any patient with altered steroid requirement within 2 weeks of MRI should be classified as “non-evaluable” at that time point for response or progression.

Further guidance on endpoints in immunotherapy trials in BM—particularly the issue of separating out the intracranial and extracranial response—has been provided by the FDA (20). Whilst criteria are dynamic (21, 22), such guidance should reduce the over-reporting of progressive disease due to imaging immune responses and consensus guidelines—which are technically low-quality evidence—will invariably will be applied in trials going forward (23).

In summary, measuring size on post-contrast T1W MRI remains a major part of assessing response to treatment for BM, including immunotherapy. Volume is likely to increase in importance compared to 2-D measurements in the future. Despite being easily understood and established in clinical practice, there are significant problems when applying size measurements alone to BM receiving immunotherapy due to the inflammatory response affecting tumor size and shape. Modern guidelines and trial criteria are reflecting this uncertainty, but ultimately more advanced imaging techniques are needed and treating clinicians and radiologists must have information on the precise timing of immunotherapy, steroids, and the patient’s clinical status to interpret the images.

## Beyond Size and Shape: Perfusion and Diffusion

Diffusion-weighted imaging (DWI) has many advantages, being a quick, reproducible, and well-studied sequence in neuro-oncology, which is available on many standard scanners including in non-academic centers. In BM in particular, DWI parameters have been widely investigated as biomarkers of response to radiation and surgery (24) and may demonstrate biological change in both the tumor and the peritumoral region (25, 26), the latter being especially important in immunotherapy response (5).

Apparent diffusion coefficient (ADC) maps can be generated from standard DWI sequences and measured in voxels, regions, or volumes of interest. Diffusion tensor imaging (DTI) generally involves more directions and/or b-values and allows fractional anisotropy (FA) maps and thence putative white matter tracts to be derived. ADC may be a surrogate of cellularity, although for BM this will vary somewhat depending on the primary cancer type (26, 27). For a BM that is continuing to progress after the start of treatment to the point of follow-up imaging, it would be broadly expected for the ADC values within the BM to decrease as the cellularity increases, and this has been shown for specific histologic types, e.g., renal cell carcinoma (28). In a BM treated with immunotherapy, intratumoral infiltration by immune cells, necrosis, and edema could complicate this picture, and no studies have reported measuring ADC in BM undergoing immunotherapy yet. There are some indicators from the glioma literature; for example, a trial of dendritic cell vaccine therapy in eight patients with glioblastoma (GBM) found the minimum tumor ADC values (but not mean values) from the contrast-enhancing regions were lower in tumors that were about to progress or had already progressed compared to those that were stable or responding (29). This highlights another issue that will be relevant for BM studies, which is that clear definitions are needed of how individual biomarkers such as the ADC of the tumor or peritumoural region are recorded. For example, a study of 19 patients with recurrent GBM found increased relative ADC within contrast-enhancing tumor regions in 86% of those responding to ICI treatment within the first 6 months (30). Relative ADC was generated by normalizing the measured ADC to the contralateral white matter. Although this is a common methodological approach in brain tumor studies, it is notable that the small number of reports so far have all used different DWI metrics (e.g., minimum ADC, fractional increased ADC, intermediate ADC volume of interest). Variability in definition is a particular problem in BM, especially when considering multiple time points and multiple small BMs. Unlike glioma, the edema around BM is also largely not infiltrated by tumor cells; therefore, data on the use of ADC readings further out from the tumor border, in the region of FLAIR signal change, are also likely to be less relevant (31). One case report in GBM notably used restriction spectrum imaging (RSI), which applies multiple b-values and gradient directions to try and separate out different components of the diffusion signal, and this may be one option to overcome heterogeneity of signal, but again this has not yet been applied to BM (32).

Perfusion-weighted imaging (PWI), like DWI, has a strong basis in preclinical studies for detecting viable tumor specifically in BM (33). In clinical practice, necrosis, edema, steroids, and anti-angiogenic therapies prior to immunotherapy may confound the measurement of PWI. Logically, one would expect increased blood flow, by whatever metric, to correlate with active tumor, and this has been investigated for patients with melanoma BM receiving ICI using two different dynamic contrast enhanced (DCE)-MRI metrics, the relative Vp90 and relative K^trans^ (34). DCE-MRI is one subtype of PWI that uses the T1 relaxation characteristics of gadolinium contrast agents to model the distribution of contrast between the vascular and interstitial space and indicates vascular permeability (for example, due to blood-brain barrier breakdown at the tumor interface). The other common PWI technique in neuro-oncology practice is dynamic susceptibility contrast (DSC)-MRI. This measures the signal loss on a T2 weighted sequence as contrast passes through the area of interest and is more informative of blood flow to a tumor.

The effects of radiation on PWI will complicate assessment further, and this is relevant since combination ICI with radiosurgery is a potentially valuable paradigm in treating BM. It has been shown that during this treatment, if there is no increase in the relative cerebral blood volume (rCBV, a PWI measure derived from DSC-MRI), this favors treatment effect over progressive tumor (35). Finally, the effects of anti-angiogenic agents on PWI will also need to be considered. A recent study of ICI in GBM found there was no predictive value of rCBV derived from DSC-perfusion or K*^trans^* derived from DCE-perfusion on treatment response; however, crucially 5/19 patients had received and continued to receive anti-angiogenic treatment during the study, with inevitable effects on PWI (30). Given that up to approximately 10–20% of patients may experience radionecrosis (36), potentially more likely in the ICI with radiosurgery-treated patients, anti-angiogenic agents like bevacizumab may be even more frequently used (37). It remains to be seen if ICI affects the tumor and peritumoral region of BM in different ways compared to vaccine or cell-based immunotherapy or if PWI may have different value in BM from primaries where neo-angiogenesis is a particular feature such as NSCLC (38).

This final point is relevant more widely to imaging biomarkers in BM. Solid organ cancers generate BM with different biological behavior, potentially with different growth patterns and vulnerabilities. It remains to be seen to what extent the immune response to BM is brain-specific or tumor type specific and therefore to what extent these imaging techniques could be generalized across BM from different solid organ cancers. The only way forward is to include multiple cancer types and stratify or limit studies to single cancer types and document the molecular subtypes (e.g., BRAF status within melanoma BM), accepting this will lead to smaller studies.

In summary, PWI and DWI are both well-established techniques in clinical practice, which can be performed rapidly and reliably without additional hardware in many cases. Post processing, however, requires more specialist expertise and specific software packages in some instances. The techniques allow qualitative understanding of whether changes in tumor size or microenvironment (e.g., peritumoural edema) are reflective of viable tumor or inflammation. Further data are needed before measures from these sequences can be reliably equated to biological changes during immunotherapy and hence treatment response. They are currently surrogates of quite crude features of the tissue, such as cellular density for DWI or vascularity for PWI, and the underlying intratumoral and peritumoural microenvironment is clearly more complex.

## The Potential of Molecular Imaging

Positron Emission Tomography (PET) imaging uses radioactive tracers to assess the metabolic and biochemical activity of tissues and is the most logical technique for assessing early treatment response in BM under immunotherapy treatment. In theory it could cut through much of the confounding effect of pretreatment and radiation effects likely to be seen in this group of patients. Availability and logistics are often challenging with this technique as radiotracers are produced on site and scans are accompanied by structural imaging in the form of CT or MRI.

Amino acid PET has advantages over the conventional F-18 fluorodeoxyglucose (FDG) tracer, particularly its background to noise ratio, and there are a small number of BM-specific studies already in the literature. One descriptive study of melanoma BM patients was conducted using F-18 flourothymidine (FLT) but only collected post-treatment data on two of five patients, making interpretation difficult (39). O-(2-[18F]fluoroethyl)-L-tyrosine (FET) has been used in neuro-oncology to assess amino acid transport in brain tumors and to distinguish immune-related treatment change from progressive disease in glioma (40). A small study of five patients applied FET-PET to those with melanoma BM receiving ICI who had progressive disease as defined by T1W contrast-enhanced MRI. The maximum tumor-to-brain ratio (TBR) of metabolic activity was calculated from the standard uptake value (SUV, a semiquantitative PET measurement of activity) map by comparing tumor ROI to the normal-appearing contralateral white matter. The TBR was higher in the progressive cases, whereas time-to-peak values were shorter. One patient with pseudoprogression could in theory have been identified by FET-PET 4 weeks earlier and continued ICI despite the contrary conventional MRI findings. To overcome the intralesional heterogeneity, this study took only the BM with the highest TBR and all the patients had been heavily pretreated, being on their 2^nd^ to 4^th^ cycle of ICI by the time of scan (41). Subsequently, a larger series of BM patients from NSCLC (n=11) and melanoma (n=29) primaries was investigated using the same technique in retrospective fashion and ROC analysis performed (42). Although this was a heterogeneous group in which radiation and targeted therapy were used as well as ICI, the mean TBR (note, not the maximum) from the most metabolically active appearing lesion was 94% specific and 70% sensitive for identifying progressive disease. Furthermore, metabolic “responders” (which the authors took as a relative reduction of 10% in the mean TBR) had a significantly longer stable clinical course (10.4 months *vs* 4 months) even when at odds with the conventional MRI assessed by RANO criteria. 11-C methionine is another tracer that has been applied to BM (43) but not in those receiving immunotherapy. The same tracer has also been used in 14 patients with GBM receiving peptide based vaccination to inform treatment changes, although it required a voxel-wise method comparing pre- and post-treatment scans (likely due to the heterogeneity of GBM), and this might be difficult with most, smaller BM (44).

At an even more detailed and personalized level, specifically engineered PET tracers can be used as *in vivo* imaging tool to look at cell trafficking, which allows the unfolding immune response to be assessed. This technique has been applied in GBM (but not BM) in seven patients treated with engineered (chimeric antigen receptor or CAR) cytotoxic T-cells. The signal detected using a probe to image the subsequent infiltration of those cells into tumor was distinct from any disease progression (45, 46). CAR-T cell immunotherapy in GBM patients has also been assayed with MR spectroscopy, although this study combined this with other markers from DWI and PWI (47).

MR spectroscopy is a longstanding technique in brain imaging that has been very sparsely applied to monitoring immunotherapy responses, in BM or in brain tumors generally. It takes time to acquire, depending on anatomical coverage and resolution, which are severely limited, and is not easily applied to BM, which are often small lesions and multifocal. Generally, a defined set of metabolites such as choline (reflecting cell membrane turnover), N-acetyl aspartate (neuronal integrity), lactate (anaerobic metabolism), and lipid (necrosis) are compared to an internal control peak such as creatine or to one another with the ratios reflecting tumor or normal tissue. Although a wide range of methods and techniques exists that are beyond the scope of this review, ultimately there is much overlap of different tissue and tumor types. An older report of two patients with intratumoral IL4 injection into GBM used the MRS finding of low choline in the context of increasing enhancement to justify continuing observation and treatment, and the tumors subsequently regressed (48). The prospects of MRI spectroscopy being widely used in BM studies of immunotherapy response are seemingly limited, and although a number of studies report using it sporadically to distinguish pseudoprogression from viable tumor in BM treated with ICI in their methods, the sensitivity and specificity are not formally described (49).

Chemical exchange saturation transfer (CEST) imaging is a more recent technique based on the chemical composition of the tissue being assessed that detects certain compounds at very low concentrations by means of exchange of protons with the surrounding water molecules (50). The technique can detect both exogenous contrast agents as well as several endogenous substances. Amide proton transfer CEST imaging uses proteins and peptides as an endogenous contrast agent and has been applied to some common neuro-oncology problems such as distinguishing solitary BM from GBM (51) and radiation necrosis from BM progression (52). CEST may have a future role in assessing response to immune therapy due to a variety of endogenous agents that can be assessed as well as a broad scope for development of exogenous agents, including “responsive” agents capable of detecting pH, ion composition, and other tissue parameters (53, 54). Amide-proton transfer has recently become available as a commercially available software option on some clinical MRI systems, but other CEST techniques remain preclinical research tools.

In summary, spectroscopy is non-invasive and involves no ionizing radiation tracer but takes time, is subject to artefacts, and is poorly studied in BM and even more poorly studied in immunotherapy. With multiple BM often being treated, it is impractical to imagine that spectroscopy will be incorporated into clinical trials of immunotherapy for BM very widely. As a result, it is hard to correlate changes in different spectral peaks with any definite clinical change and to distinguish viable tumor from inflammation as there will not be a bank of data to analyze. PET imaging is only available in specialist centers and may require more tailored radioactive tracers to assess responses to immunotherapy, which makes the prospect of routine clinical use very distant. Nonetheless, the centers using immunotherapy for BM are likely to be specialist oncology departments and have ready access to PET compared to the community. Particularly, FET-PET does appear to demonstrate clear ability to assess the response of BM to immunotherapy regardless of changes in other MRI parameters.

All these techniques offer the tantalizing prospect of assessing the early response to therapy; however, perhaps a more pressing need is the development of imaging biomarkers that will predict treatment response before even starting therapy (8).

## Biomarkers to Predict Response

In large clinical trials to date, the response of BM to immunotherapy, especially ICI, is heterogenous, and those patients who do not respond may experience significant toxicity or adverse events. There is a clear clinical need for biomarkers that can predict the subsequent response to immunotherapy. The most logical way of doing this for ICI in BM would be demonstrating an immunologically favorable microenvironment before commencing treatment (4, 5).

### Conventional Anatomic Imaging

Structural or anatomical imaging may have some value in this regard, in that T2 and FLAIR sequences can quantify the degree of peritumoral edema and inflammation. This varies greatly with the number and location of BM, the use of steroids, and the timing of any radiation treatments. Nonetheless, an analysis of 116 BM by conventional MRI and immunohistochemistry of the resected tumors found that the density of CD8+ tumor-infiltrating lymphocytes was correlated with the volume of peritumoral edema on preoperative MRI (55). Notably, these were all solitary BM, steroids did not seem to make any difference, and edema was graded in a novel fashion by radiologists, scoring its extent from the tumor margin on T2W images, <1 cm, >1 cm not crossing the midline and >1 cm crossing the midline. They suggest edema may be a surrogate marker of the immune response pretreatment in BM, but this needs to be more quantitatively investigated—most logically as a volume of interest on FLAIR and T2W images—and recorded longitudinally in BM patients receiving immunotherapy, for example. It is was initially suggested that the interaction of radiation and ICI can cause a temporary increase in size and edema where SRS was given prior to ICI in some patients, but other studies with the same histology and agents have not reproduced this, showing instead a gradually declining volume of edema and tumor with response (56, 57). A recent paper used a mathematical model of immunotherapy efficacy based on conventional anatomic imaging to examine the response to ICI (ipilimumab and nivolumab) amongst patients with BM from different clinical trials (58). The BM growth rate at first restaging was as accurate as the retrospective determination of immune response at predicting response, and no additional imaging beyond the clinical structural scans were used. Ultimately, many terms in the model such as intrinsic growth rate of the tumor were determined from previous scans, but in the future, it might be possible to infer this from tissue or blood analysis. The advantage of conventional structural imaging is that it can be repeated quickly and reliably at multiple time points and analysis can potentially be automated, even if size and shape are not very specific.

### Radiomics

**Figure 2:**
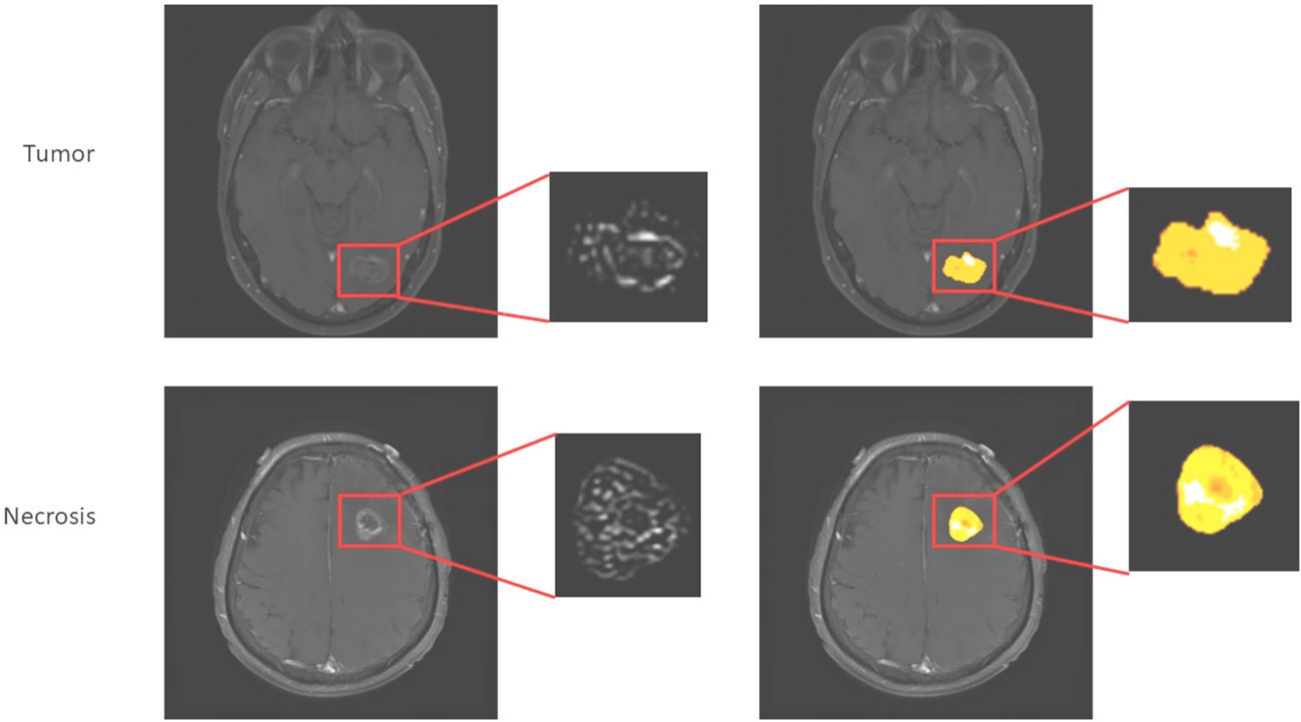
Application of radiomics approach to melanoma brain metastases to distinguish tumor from necrosis/treatment effect (unpublished work, DM). This is similar to the approach used in (61), which found higher complexity in edge-filtered images of the sort seen on the left as well as higher entropy illustrated by various extracted features such as shown on the right in progressing BM after immunotherapy *versus* responding.

One emerging method of deriving more information from conventional structural imaging is radiomics. This is a computational method for extracting many (potentially hundreds of) image features related to texture and shape. Multivariable regression or other machine learning techniques can then be used to develop a classification or prognostic model. Radiomics can be applied to any form of imaging, including both conventional anatomical imaging and DWI, PWI, and combinations of multiple modalities. Radiomics has already been used in extracranial disease (59) and melanoma BM (60). In the latter, pretreatment post-contrast T1W scans were manually assessed by a radiologist and target lesions segmented using freely available software (ITK-SNAP). Several features were associated with overall—although not progression-free—survival, and whilst these did not hold up in multivariate analysis, a Laplacian of Gaussian (LoG) feature was significant in a validation cohort, suggesting there was a biological signal. This technique has the advantage of not needing additional sequences or tracers and so could in theory be widely used, including retrospectively, e.g., in case-control or retrospective cohort studies. An illustration of this technique is given in **Figure 2**. There is limited evidence of repeatability and reproducibility of radiomics results, as well as limitations in the quality of reporting in the literature (62). As a result of these concerns, consensus guidelines and definitions have been proposed, with the aim of improving the quality of reporting (63).

### Diffusion

**Figure 3:**
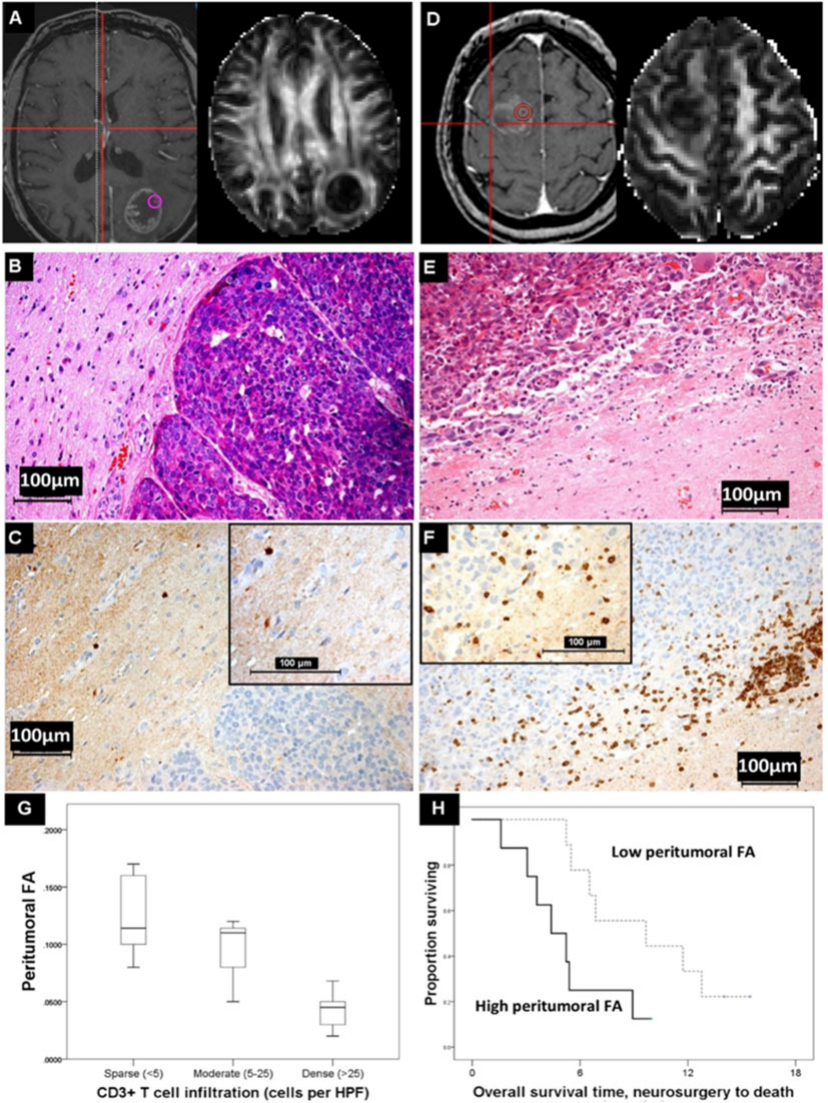
Diffusion MRI changes and immune cell infiltration. **(A)** A lung adenocarcinoma brain metastasis which shows little peritumoural white matter disruption and has a high peritumoural fractional anisotropy (FA) value at the biopsy location shown. **(B)** H&E, **(C)** CD3-stained serial section showing sparse T cell infiltration in this same region (inset, magnified). This contrasts with the breast cancer brain metastasis in **(D)**, where there is more change in the peritumoural white matter and the FA value in the peritumoural region shown is lower. Here, there is dense peritumoural T cell infiltration [**(E, F)** and inset, magnified]. **(G)** The cases with a peritumoural FA >median (n=8, thick line) died sooner after neurosurgical resection of their metastasis than those with a lower peritumoural FA (n=9, dashed line) (log rank statistic = 4.566, p<0.05). **(H)** The FA values differentiated categories of peritumoural CD3+ T cell density (<5, 5–25, and >25 per high-power field) in the co-localized image-guided biopsy regions (Kruskal-Wallis, p<0.05), although such a relation is not seen with other immune cells such as CD68+ macrophages or CD20+ B cells, nor is it seen with other MRI measures, such as the mean diffusivity (MD). Reproduced by Creative Commons license from (65).

Focusing on the peritumoral region but moving to advanced imaging, a series of 18 BM being removed surgically was investigated using image-guided samples from the peritumoral region, and a higher density of tumor-infiltrating lymphocytes was associated with prolonged overall survival regardless of primary. Additionally, higher CD3+ T cell density was also associated with a reduction in peritumoral FA, a measure of diffusion that is a surrogate of white matter tract integrity and has been widely investigated in other neuroinflammatory pathologies such as multiple sclerosis and radiation injury. This implies that the BM microenvironment could be assessed non-invasively, and studies are underway to determine if this is a biomarker of response to subsequent ICI in melanoma (64, 65). These results are illustrated in **Figure 3**. As with all BM, there is evidence of discordant mutations between the metastasis and the primary (66), and the impact of BM-specific changes—such as BRAF mutations in metastatic melanoma—on the imaging responses must also be investigated.

### Molecular Imaging

Since PD-L1 expression correlates with response to ICI with targeted treatment, PET imaging with an engineered tracer has been used in patients with non-small-cell lung cancer to assay this. Uptake correlated with tumor positivity for PD-L1 at immunohistochemistry analysis and treatment response to the ICI agent nivolumab (61). Two of the 13 patients in this study had untreated BM, and both patients—but not all their BM—showed intracranial uptake, albeit with lower SUV values than in extracranial lesions. This is important as we know that due to the branched evolution of BM, the extracranial disease may not indicate the same susceptibilities to treatment as intracranial metastases (66).

Probes have been developed that are even more specific to the immune response and applied in other brain tumors but not yet in BM. 18-F CFA is a probe specifically developed to accumulate in proliferating T cells and was used in two patients to demonstrate immune activation after dendritic cell vaccination (67). Similarly, the effectiveness of vector-mediated HSV-1-tk gene expression in a phase I/II gene therapy trial for GBM was measured using a specific PET tracer ([124I]-FIAU) and correlated with therapeutic response (68).

The advantage of molecular imaging is thus that immunotherapy-specific tracers can be developed but with the problem that ever more specific tracers are harder to produce, less widely applicable, and less well studied.

## Summary

Most of the published literature on imaging biomarkers of immunotherapy for brain tumors relates to glioma, particularly recurrent GBM, as these are the types of cases that enter such clinical trials. BM are an increasing target for such therapeutics, and novel biomarkers and techniques are needed to overcome the unique challenges in this disease. This includes the interaction of the BM with the native brain microenvironment, which is likely to vary for metastases from different primaries, as well as the differential intra- and extracranial disease responses to be assessed. The pros and cons of each technique are listed in **Table 1** and summarized below:

- Structural imaging will remain important—size, and ultimately volume, continues to be a crude marker of early response but not any indication before treatment. The radiomics approach may have use in incorporating large amounts of existing clinical imaging data in a useful manner.
- Physiological imaging is the most applicable and available advanced technique, diffusion is promising and well-studied, whilst perfusion also appears to reliably associate with tissue characteristics during treatment. These sequences are often included in the BM workup and treatment workflow so could be excellent for finding early markers after therapies start to affect the tumor tissue. In the peritumoral regions, such techniques may indicate an immune active microenvironment and could be a pretreatment marker.
- Molecular markers are highly specific, and many BM patients have PET studies as part of staging investigations, so this is an opportunity for investigating and defining pretreatment biomarkers. Tracers to look at the various ICI-targeted pathways are being developed and will need to be used in trials for intra- and extracranial disease with a variety of primary tumors.

**Table 1:** Summary of the current imaging techniques for predicting and measuring the response to immunotherapy in brain metastases.

Imaging method	Metrics investigated	Advantages	Disadvantages	Technique feasibility
Conventional MRI	Tumor diameter, volume from T1W post-contrast sequences.	Quick, reproducible, widely available including non-specialist centers. Well-established guidelines for interpretation. Can potentially be integrated into radiomics models or automated, AI pipelines.	Rely on operator to take measurements if not automated, volumetric imaging is not widespread, very crude surrogate of biological behaviour.	Routine clinical use.
	Peritumoral edema from T2, FLAIR sequences.			
Diffusion MRI	ADC	Fast.	Cellularity may change due to tumor or inflammatory cells.	Routine clinical use in neuro-oncology and neurology.
	FA	Peritumoral changes may have prognostic value.
	Restriction spectrum measures	May pick up more heterogeneity in future.	Novel technique with limited data. Software not readily available.	Preclinical research technique.
Perfusion MRI	CBV from dynamic contrast susceptibility MRI	Widely used in clinical practice, large amount of data on relation of perfusion to BM including from different primaries, high spatial resolution.	Post-processing software and expertise needed. May be confounded by radiation, use of anti-angiogenic agents. Unclear how blood flow relates to cellular inflammation and BBB around tumor.	Routine clinical use in neuro-oncology.
	K^trans^ from dynamic contrast-enhanced MRI.			Routine clinical use in oncology for specific tumor types (e.g., breast, prostate), occasional use in neuro-oncology.
MR spectroscopy	Choline/creatine ratio	Well established in brain imaging, multivoxel in addition to single-voxel imaging improves spatial discrimination.	Time-consuming, poor spatial resolution given size of lesions in brain metastasis cases, not specific for inflammation *versus* viable tumor	Routine clinical use in neuro-oncology and other neurological conditions.
CEST MRI	Amide-proton transfer	Less confounded by radiation.	Limited availability	Translational research/early clinical technique.
		No contrast agent/tracer required.	Limited data
			Time-consuming
	Others	Wide research scope, including sensitivity to multiple endogenous metabolites and large variety of molecular tracers.	Little data.	Preclinical research technique.
PET	Various tracers, 18F-FET PET most widely studied	Not confounded by radiation, steroids. Combined with structural/conventional imaging. PD-L1 specific tracer may predict response to immune checkpoint inhibition.	Poor availability due to need to produce specific tracers and expertise, poor spatial resolution.	Specialist clinical technique.

## Future Directions

There is a clear need for further investigation of imaging biomarkers of immunotherapy in BM; these may develop along with extracranial imaging techniques for assessing other metastases or arise from the existing intracranial techniques for assessing glioma. These may be novel sequences or probes or composites of existing ones. All must account for the unique brain microenvironment and the intralesional variation in response.

## Author Contributions

RZ and MJ devised the article and wrote the outline. RZ wrote the first draft; and CP, SM, DM, CC, and MR added specific sections and figures. All authors contributed to the article and approved the submitted version.

## Funding

RZ receives funding from Cancer Research UK (C68514/A28190) and the Royal College of Surgeons of England (Pump Priming Grant for new investigators 2019).

## Conflict of Interest

The authors declare that the research was conducted in the absence of any commercial or financial relationships that could be construed as a potential conflict of interest.

## Publisher’s Note

All claims expressed in this article are solely those of the authors and do not necessarily represent those of their affiliated organizations, or those of the publisher, the editors and the reviewers. Any product that may be evaluated in this article, or claim that may be made by its manufacturer, is not guaranteed or endorsed by the publisher.

## Abbreviations

ADC, apparent diffusion coefficient; BM, brain metastases; CAR, chimeric antigen receptor; CBF, cerebral blood flow; CBV, cerebral blood volume; DCE-MRI, dynamic contrast enhanced MRI; DSC-MRI, dynamic susceptibility contrast MRI; DTI, diffusion tensor imaging; DWI, diffusion-weighted imaging; FA, fractional anisotropy; FDG, F-18 fluorodeoxyglucose; FLT, F-18 flourothymidine; FET, O-(2-[18F]fluoroethyl)-L-tyrosine; FLAIR, fluid attenuated inversion recovery; GBM, glioblastoma multiforme; ICI, immune checkpoint inhibitors; MD, mean diffusivity; NSCLC, non-small-cell lung cancer; PET, positron emission tomography; PWI, perfusion-weighted imaging; ROI, region of interest; RSI, restriction spectrum imaging; SUV, standard uptake value; T1W, T1 weighted; T2W, T2 weighted; TBR, tumor-to-brain ratio; VOI, volume of interest.
